# Effects of brief sodium fluoride treatments on the growth of early and mature cariogenic biofilms

**DOI:** 10.1038/s41598-021-97905-0

**Published:** 2021-09-14

**Authors:** Ye Han

**Affiliations:** Department of Preventive Dentistry, School of Dentistry, Institute of Oral Bioscience and BK 21 Plus Program, Jeonbuk National University, Jeonju, 561-756 Republic of Korea

**Keywords:** Microbiology, Bacteria, Biofilms, Microbial communities

## Abstract

Although fluoride has been widely used as a preventive agent for dental caries, the effects of fluoride on the activities of biofilms in different stages of cariogenic biofilm formation are less studied. This study was designed to investigate the antibiofilm activity of sodium fluoride during the early and mature stages of *Streptococcus mutans* (*S. mutans*) biofilm formation. *S. mutans* biofilms were formed on saliva-coated hydroxyapatite disks. In the early (0–46 h) and mature (46–94 h) biofilm stages, the biofilms were treated with different concentrations of fluoride (250, 500, 1000, 2000 ppm; 5 times in total, 1 min/treatment). Acidogenicity, dry weight, colony-forming units (CFUs), water-soluble/insoluble extracellular polysaccharides (EPSs), and intracellular polysaccharides were analysed, and confocal laser scanning microscopy images were obtained of the two stages of biofilms to determine antibiofilm activities of fluoride at varying concentrations during the formation of early and mature biofilms. In the early stages of cariogenic biofilm formation, test groups with all fluoride concentrations significantly inhibited the growth of *S. mutans* biofilms. The antibiofilm and anti-EPS formation activities of the brief fluoride treatments increased with a concentration-dependent pattern. At the mature biofilm stage, only the 2000 ppm fluoride treatment group significantly inhibited biofilm accumulation, activity, and intracellular/extracellular polysaccharide content compared with those of the control and other fluoride treatment groups. The antimicrobial effect of fluoride treatment on the growth of *S. mutans* biofilms was linked with the stage of cariogenic biofilm formation. The inhibition of *S. mutans* biofilm growth by fluoride treatment was easier in the early formation stage than in the mature stage. Fluoride treatment in the early stage of cariogenic biofilm formation may be an effective approach to controlling cariogenic biofilm development and preventing dental caries.

## Introduction

Dental caries is an oral disease associated with dental biofilms^1^. The increased levels of acidogenic and aciduric bacteria in microorganism are of great significance in the pathogenesis of dental caries^1^. Through glycolysis, acidogenic bacteria reduce the pH of dental biofilms developed on the surfaces of teeth exposed to dietary sugar^1^. The low pH environment further accelerates the growth of acidic and acidogenic bacteria, including *mutans streptococci*, other *acidogenic streptococci*, *lactobacilli*, and *bifidobacteria*^2^. The compositions of cariogenic biofilm microflora are complex^3^. Although there may be other acidogenic and aciduric bacteria^4^, *S. mutans* is considered one of the important aetiological agents for the development of dental caries^5,6^. *S. mutans* can produce glucosyltransferases and synthesize extracellular polysaccharides with sucrose as a substrate. EPSs contribute to bacterial adhesion on the tooth surface, colonization, and formation of plaque biofilms, which are important cariogenic factors of *S. mutans*^7^. In addition, acid production through carbohydrates and acid tolerance at low pH are also the major cariogenic factors of *S. mutans* that result in the loss of local hard tissues and initiation of the cariogenic process^7^. Decreases in *S. mutans* adhesion to tooth surfaces, acid production, and EPS formation by *S. mutans* biofilms may reduce the incidence of dental caries.

Fluoride is a widely used preventive agent for dental caries. Fluoride products, such as fluoride water, mouthwash, ordinary toothpaste, and prescription toothpaste, play an important role in preventing dental caries^8^. Reducing enamel solubility and promoting its remineralization are considered the underlying mechanisms of fluoride action against dental caries^9,10^. Previous findings have shown that fluoride inhibits the adhesion of *S. mutans* to the surface of hydroxyapatite^11^ and also inhibits acid production, acid tolerance, and glucosyltransferase production of *S. mutans* biofilms^12–14^. In addition, the effect of fluoride on cariogenic biofilm virulence has been identified. Even one 1-min fluoride treatment with a minimum concentration of 300 ppm can affect the cariogenic characteristics of *S. mutans* biofilms^15^, although it cannot sustain antiviral activity because of the recovery of *S. mutans* from fluoride shock over time^16^. Periodic 1-min fluoride treatments, however, reduce the dry weight and acid production of *S. mutans* biofilms in a concentration-dependent manner^17^. A growing number of studies have focused on the mechanism by which fluoride inhibits the cariogenic biofilm growth of *S. mutans*. Nevertheless, the relationship between the anticariogenic biofilm activity of fluoride and the growth stages of biofilms as well as the effect of brief fluoride treatments on the virulence of early and mature cariogenic biofilms remain largely unclear. It is necessary to validate the hypothesis that brief fluoride treatments yield anticariogenic biofilm activity, mainly through the early stage of biofilm formation. Therefore, this study is designed to demonstrate the effects of fluoride on the development of *S. mutants* biofilms that depend on the biofilm formation stage. In brief, *S. mutans* biofilms were treated with different concentrations of fluoride (0, 250, 500, 1000, 2000 ppm) in the following two stages: early biofilm formation stage at 0–46 h and mature biofilm stage at 46–94 h. The effects of varying concentrations of fluoride on the development of *S. mutans* biofilms in the two stages were compared.

## Result

### Effects of brief fluoride treatments during early *S. mutans* biofilms formation

#### The microbiological and biochemical analyses in the early *S. mutans* biofilms formation

**Figure 1:**
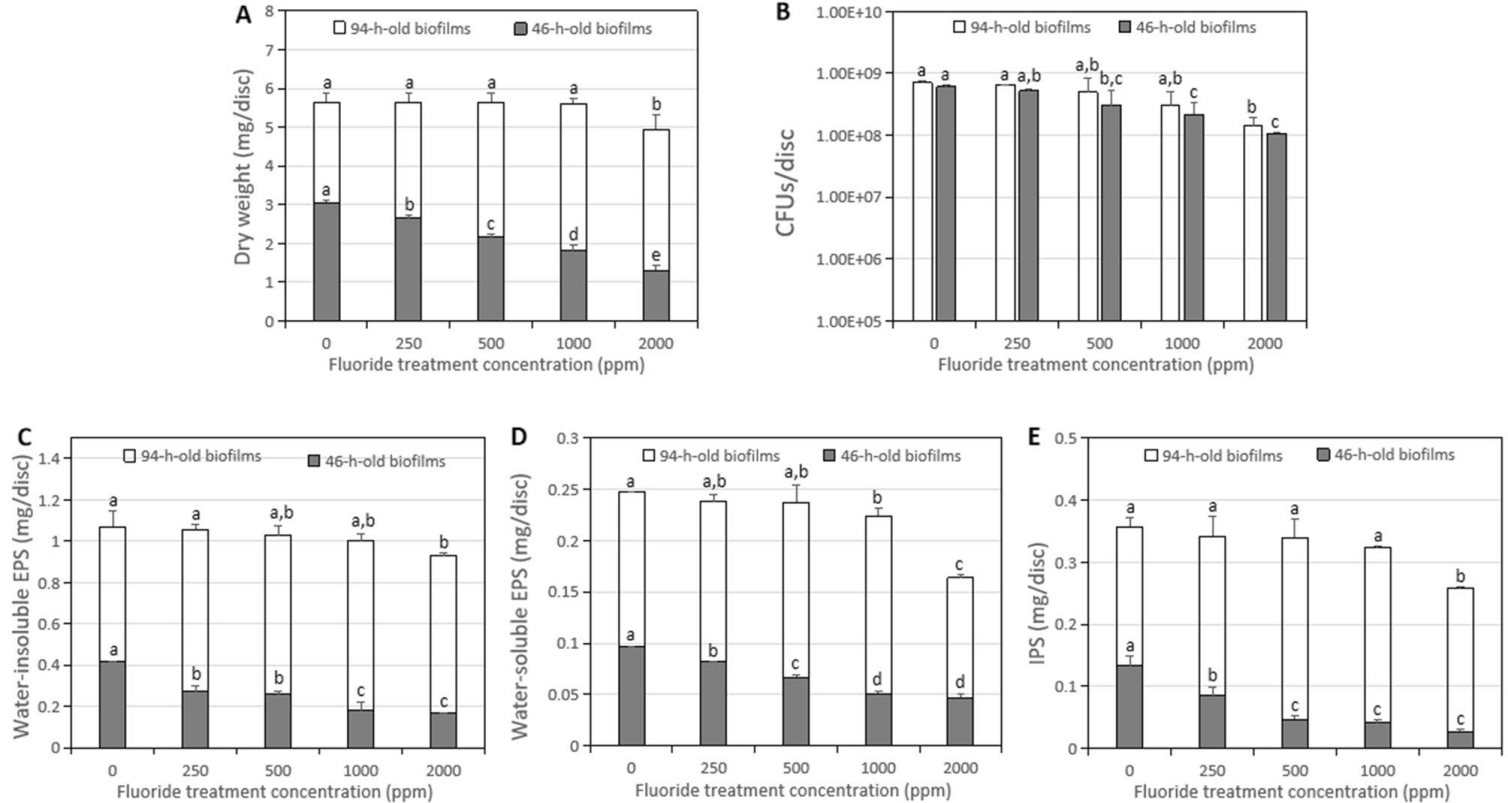
Change in the microbiological and biochemical composition of the 46/94-h-old *S. mutans* biofilms treated with different concentrations of fluoride. (**A**) Dry weight. (**B**) CFUs. (**C**) Water-insoluble EPSs. (**D**) Water-soluble EPSs. (**E**) Intracellular polysaccharides (IPS). Data represent mean ± standard deviation. In the 46-h-old *S. mutans* biofilms, (**A**–**E**), *p* < 0.05. In the 94-h-old *S. mutans* biofilms, figure (**A**–**C**), *p* > 0.05; (**D**,**E**), *p* < 0.05. *p < 0.05: significantly different from each other. *p* > 0.05: values followed by the same superscript are not significantly different from each other.

As shown in Fig. 1, brief fluoride treatments with all tested concentrations significantly reduced the dry weight accumulations, bacterial activities, and polysaccharides formation of 46-h-old *S. mutans* biofilms in a concentration-dependent manner, and the differences between each treatment group and the control group were statistically significant (Fig. 1A–E; *p* < 0.05). The lowest values were found for the 2000 ppm fluoride treatment group.

### Relationship between fluoride concentration and acid production of early *S. mutans* biofilms

**Figure 2:**
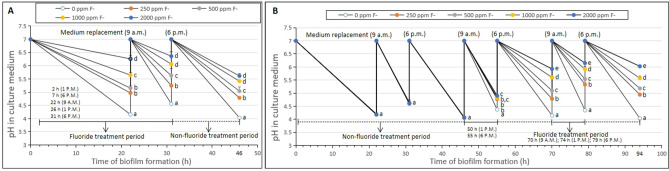
Change in the pH values of old culture medium treated with different concentrations of fluoride during 46/94-h-old *S. mutans* biofilms formation. (**A**) 46-h-old *S. mutans* biofilms (*p* < 0.05). (**B**) 94-h-old *S. mutans* biofilms (22, 31, 46 h, *p* > 0.05; 55, 70, 79, 94 h, *p* < 0.05). Data represent mean ± standard deviation. *p < 0.05: significantly different from each other. *p* > 0.05: values followed by the same superscript are not significantly different from each other.

**Figure 3:**
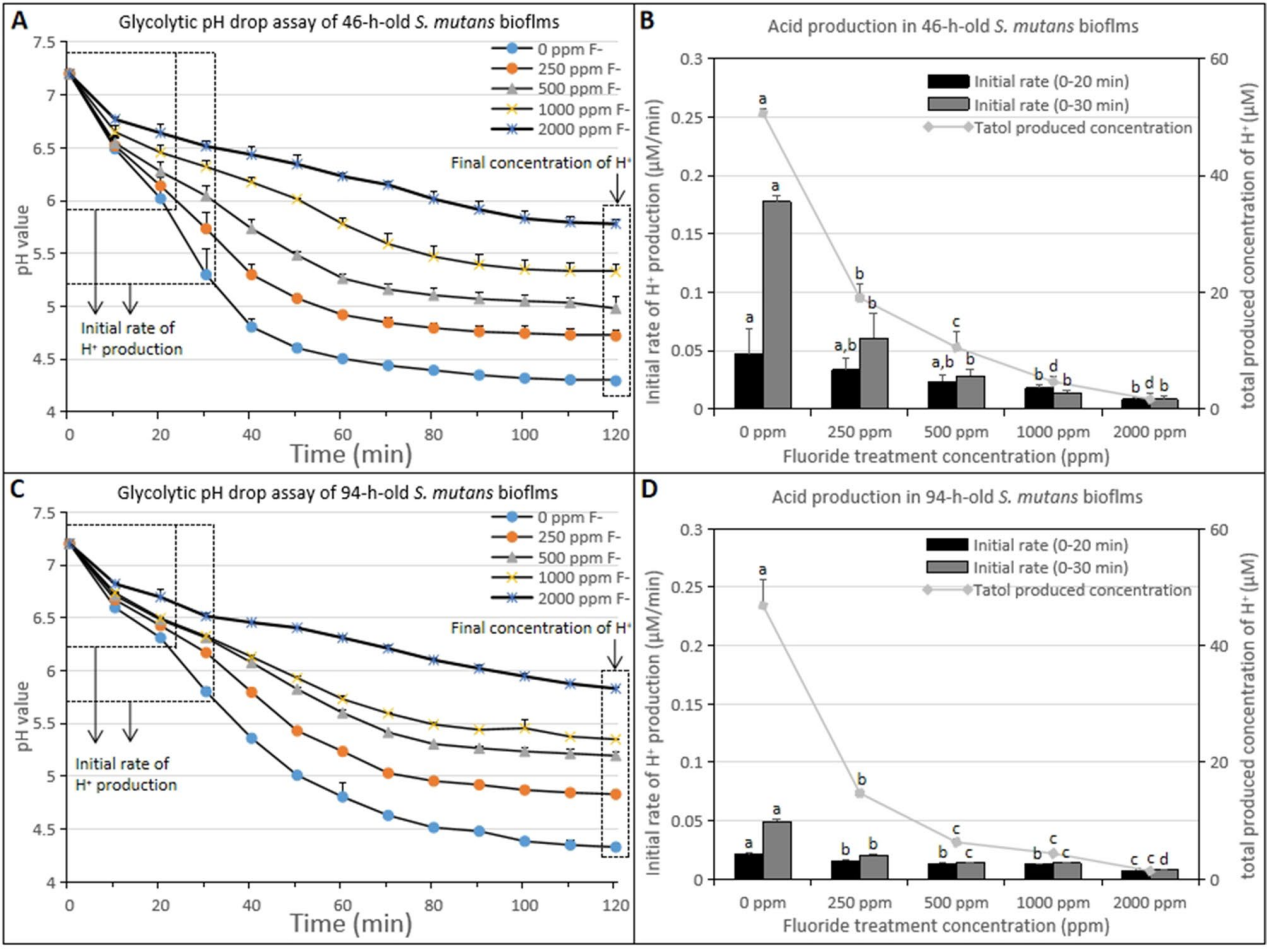
The effect of different concentrations of fluoride treatment on the acidogenicity of 46/94-h-old *S. mutans* biofilms cells. Changes in the initial rate of H^+^ production (0–20 min; 0–30 min) and total produced concentration of H^+^ (120 min) in 46/94-h-old *S. mutans* biofilms, calculated from biofilm glycolytic pH drop assay data. (**A**) Change in acid production of 46-h-old *S. mutans* biofilms (*p* < 0.05). (**B**) Initial rate of H^+^ production (0–20 min; 0–30 min) and total produced concentration of H^+^ (120 min) in 46-h-old *S. mutans* biofilms (*p* < 0.05). (**C**) Change in acid production of 94-h-old *S. mutans* biofilms. (**D**) Initial rate of H^+^ production (0–20 min; 0–30 min) and total produced concentration of H^+^ (120 min) in 94-h-old *S. mutans* biofilms (*p* < 0.05). Data represent mean ± standard deviation. *p < 0.05: significantly different from each other.

Figure 2A showed the pH changes of the old culture medium, it was found that all tested concentrations of fluoride treatment groups inhibited the acid-producing capacity of the biofilm in the early formation stage of the biofilm (*p* < 0.05). The concentration of acid in the old medium decreased in a concentration-dependent manner, and the highest inhibition ability was shown for the 2000 ppm fluoride treatment group. The glycolytic pH drop assay was used to determine the effect of brief fluoride treatment on the acid production and acid tolerance of 46-h-old *S. mutans* biofilms. As shown in Fig. 3A,B, brief fluoride treatments inhibited acid production in the early stage of cariogenic biofilms. The acidification activities of biofilms were inhibited in all fluoride treatment groups, and the 2000 ppm fluoride treatment group showed the lowest initial rate and total acid production (Fig. 3B). Additionally, the 2000 ppm fluoride treatment group showed reduced acid resistance of cariogenic biofilm cells, and it showed the highest final H^+^ concentration, indicating the most pronounced effect on the acid resistance of biofilm cells (Fig. 3A).

### Confocal laser scanning microscope study of early *S. mutans* biofilms

**Figure 4:**
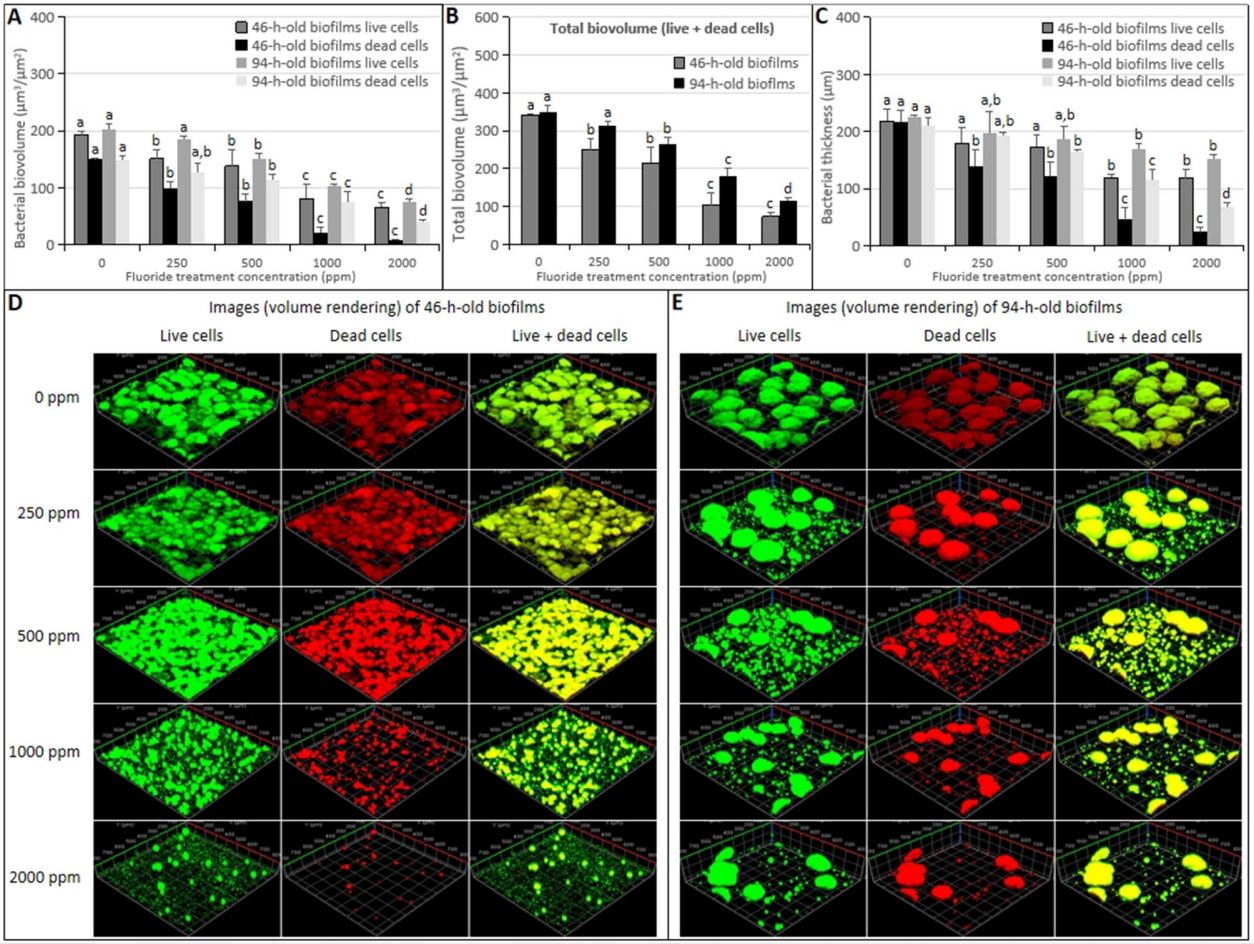
Change in confocal laser scanning microscopy of bacterial cells in the 46/94-h-old *S. mutans* biofilms treated with different concentrations of fluoride. (**A**) Bacterial biovolume. (**B**) Total biovolume (live + dead cells). (**C**) Bacterial thickness. (**D**) Representative confocal images of 46-h-old *S. mutans* biofilms. (**E**) Representative confocal images of 94-h-old *S. mutans* biofilms. Data represent mean ± standard deviation. Significantly different from each other (*p* < 0.05).

**Figure 5:**
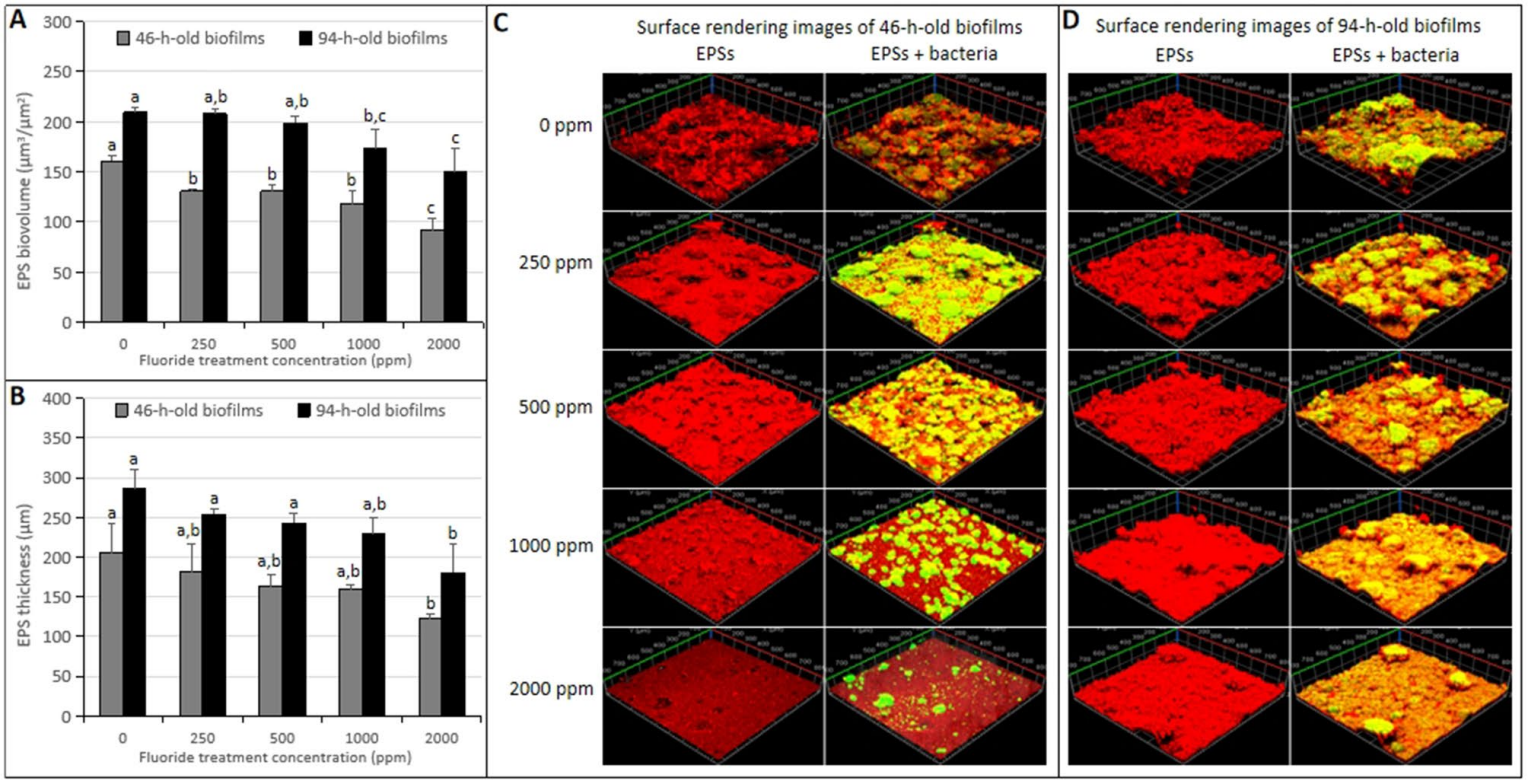
Change in confocal laser scanning microscopy of EPSs in the 46/94-h-old *S. mutans* UA159 biofilms treated with different concentrations of fluoride. (**A**) EPS biovolume. (**B**) EPS thickness. (**C**) Representative confocal images of 46-h-old *S. mutans* biofilms. (**D**) Representative confocal images of 94-h-old *S. mutans* biofilms. Data represent mean ± standard deviation. Significantly different from each other (*p* < 0.05).

To further evaluate the effect of brief fluoride treatment on early *S. mutans* biofilms formation components and structure, the confocal laser scanning microscopy analysis was performed. As shown in Fig. 4, the bacterial biovolumes of live/dead cells, bacterial thicknesses, and total biovolumes of the 46-h-old *S. mutans* biofilms were reduced by brief fluoride treatments in a concentration-dependent manner. The lowest values were found for the 2000 ppm fluoride treatment group (Fig. 4A–C; *p* < 0.05). Significant differences in the bacterial biovolumes of live/dead cells, bacterial thicknesses, and total biovolumes of the 46-h-old *S. mutans* biofilms were detected between each treatment group and the control group. The three-dimensional image of bacterial microcolonies of 46-h-old *S. mutans* biofilms showed that with the increased concentration of the brief fluoride treatment, the morphology was changed to reflect a lower number of the live/dead cells microcolonies with smaller sizes and looser arrangements (Fig. 4D). Brief fluoride treatments also affected EPS formation of *S. mutans* biofilms, as shown in Fig. 5A,B. Brief fluoride treatments reduced EPS biovolumes (*p* < 0.05) and thicknesses (*p* > 0.05). With the increased concentration of the brief fluoride treatment, the anti-EPS formation increased in a concentration-dependent manner. EPSs and bacterial microcolony images of 46-h-old *S. mutans* biofilms showed minimal concentrations of homogeneous structures of EPSs covering and surrounding bacterial microcolonies for the 2000 ppm fluoride treatment group (Fig. 5C).

### Effects of brief fluoride treatments during mature *S. mutans* biofilms formation

The microbiological and biochemical analyses in the mature *S. mutans* biofilms formation. As shown in Fig. 1, only the treatment group with 2000 ppm fluoride presented significant inhibition of dry weight accumulation, bacterial activity, and polysaccharides formation of the 94-h-old *S. mutans* biofilms compared with those of the control and other fluoride treatment groups (Fig. 1A–C, p > 0.05; Fig. 1D,E, p > 0.05). The lowest values were found for the 2000 ppm fluoride treatment group.

### Relationship between fluoride concentration and acid production of mature *S. mutans* biofilms

Figure 2B showed the pH changes of the old culture medium, it was found that all tested concentrations of fluoride treatment groups inhibited the acid-producing capacity of the biofilm in the mature stage of the biofilm (*p* < 0.05). The concentration of acid in the old medium decreased in a concentration-dependent manner, and the highest inhibition ability was shown for the 2000 ppm fluoride treatment group. As shown in Fig. 3C,D, brief fluoride treatments inhibited acid production in the mature stage of cariogenic biofilms. The acidification activities of biofilms were inhibited in all fluoride treatment groups, and the 2000 ppm fluoride treatment group showed the lowest initial rate and total acid production (Fig. 3D). Additionally, the 2000 ppm fluoride treatment group showed reduced acid resistance of cariogenic biofilm cells, and it showed the highest final H^+^ concentration, indicating the most pronounced effect on the acid resistance of biofilm cells (Fig. 3C).

### Confocal laser scanning microscope study of mature *S. mutans* biofilms

As shown in Fig. 4, the bacterial biovolumes of live/dead cells, bacterial thicknesses, and total biovolumes of 94-h-old *S.mutans* biofilms were reduced by brief fluoride treatments in a concentration-dependent manner. The lowest values were found for the 2000 ppm fluoride treatment group (Fig. 4A–C; *p* < 0.05). Significant differences in 94-h-old *S. mutans* biofilms of the treatment group and the control group were only detected for treatment by fluoride with a minimum concentration of 500 ppm. Three-dimensional images showed that the live/dead cells in the 94-h-old biofilms showed larger microcolonies than those of the 46-h-old biofilms and uniform morphologies and compact arrangements. In addition, the number of live/dead cells microcolonies decreased and the arrangement of microcolonies became loose with the increased concentration of fluoride treatment, but the morphologies of microcolonies did not change significantly, and microcolonies in each treatment group were similar to those of the control group (Fig. 4E). Brief fluoride treatments also affected EPS formation of *S. mutans* biofilms, as shown in Fig. 5A,B. Brief fluoride treatment reduced EPSs biovolumes and thicknesses (*p* < 0.05). With the increased concentration of the brief fluoride treatment, the anti-EPS formation increased in a concentration-dependent manner. EPSs and bacterial microcolony images of 94-h-old *S. mutans* biofilms showed minimal concentrations of homogeneous structures of EPSs covering and surrounding bacterial microcolonies for the 2000 ppm fluoride treatment group (Fig. 5D).

## Discussion

Multiple pieces of evidence have supported the proposal that fluoride affects acidogenicity, acid tolerance, and EPS formation of cariogenic biofilms, such as *S. mutans* biofilms^18–20^. However, the effects of fluoride on the biological activity of oral microorganisms and biofilm activity at different stages of cariogenic biofilm formation have rarely been studied. In the present study, the *S. mutans* biofilm model was used to explore the effects of different concentrations of fluoride on the growth, virulence (EPSs and acidogenicity), and activity of early and mature *S. mutans* biofilms. In fact, the *S. mutans* biofilm model is unable to precisely mimic the complex microbial community observed in dental biofilms, but monospecies biofilms are advantageous in examining the mechanisms of *S. mutans* in biofilms with sufficient performance to validate data reproducibility and reduce variance^10,21,22^.

Biofilms are typically characterized by dense, highly hydrated clusters of bacterial cells. Bacterial adhesion and early biofilm formation are the first steps of cariogenic biofilm formation^23,24^. In the early stage of biofilm formation, the biovolumes and average thicknesses of bacterial microcolonies and EPSs of biofilms continuously increase^25^. At the mature biofilm stage beginning after 46 h, the number of CFUs and acid production capacity of the biofilm remained stable, as did the bacterial microflora of the biofilm and the biovolume and average thickness of the EPSs. The standard deviation of the biovolume and mean biofilm thickness of bacteria or EPSs remained unchanged over time^25^. Mature biofilms have thicker and more complex biofilm structures, which may affect the diffusion and penetration of antibacterial agents^26,27^. Therefore, the antimicrobial membrane activity of *S. mutans* treated briefly with fluoride during biofilm formation was studied here. As shown in Fig. 1, brief fluoride treatments with all tested concentrations significantly reduced the dry weight accumulations and bacterial activities of 46-h-old *S. mutans* biofilms in a concentration-dependent manner, and the differences between each treatment group and the control group were statistically significant (Fig. 1A; *p* < 0.05). Notably, the activity of biofilm bacteria was significantly affected in the experimental groups treated with fluoride at a minimum concentration of 500 ppm compared with that of the control group (Fig. 1B; *p* < 0.05). However, only the treatment group with 2000 ppm fluoride presented significant inhibition of dry weight accumulation and bacterial activity in the 94-h-old *S. mutans* biofilms compared with those of the control and other fluoride treatment groups (Fig. 1A,B; *p* > 0.05).

As shown by confocal laser scanning, the bacterial biovolumes of live/dead cells, total biovolumes, and bacterial thicknesses of the 46-h-old and 94-h-old *S. mutans* biofilms were reduced by brief fluoride treatments in a concentration-dependent manner. The lowest values were found for the 2000 ppm fluoride treatment group. Significant differences in the bacterial biovolumes of live/dead cells, total biovolumes, and bacterial thickness of the 46-h-old *S. mutans* biofilms were detected between each treatment group and the control group. However, significant differences in 94-h-old *S. mutans* biofilms of the treatment group and the control group were only detected for treatment by fluoride with a minimum concentration of 500 ppm (Fig. 4A–C; *p* < 0.05). The morphology of live/dead cell microcolonies of the 46-h-old *S. mutans* biofilms was uniform and tightly arranged in the control group. With the increased concentration of the brief fluoride treatment, the morphology was changed to reflect a lower number of live/dead cell microcolonies with smaller sizes and looser arrangements. High-concentration fluoride treatments at the early stage of *S. mutans* biofilms significantly affected the structural integrities of *S. mutans* biofilm cells (Fig. 4D). The live/dead cells in the 94-h-old biofilms showed larger microcolonies than those of the 46-h-old biofilms and uniform morphologies and compact arrangements. In addition, the number of live/dead cell microcolonies decreased and the arrangements of microcolonies became looser with the increased concentrations of fluoride treatment, but the morphologies of microcolonies did not change significantly, and microcolonies in each treatment group were similar to those of the control group (Fig. 4E). It was concluded that brief fluoride treatment was closely related to antibacterial biofilm activity, which was determined by the biofilm formation stage and the concentration of the fluoride treatment. Biofilms in early formation stages were more susceptible to the influence of fluoride than those in mature stages. The antibacterial biofilm activities of biofilms in the high-concentration fluoride treatment group were higher than those of the low-concentration fluoride treatment groups. According to the results of live/dead cell microcolonies, brief fluoride treatments did not have a bactericidal or killing effect on the growth of *S. mutans* biofilms. It has been reported that fluoride treatments with concentrations of 3040–5700 ppm or > 5000 μg/ml had bactericidal effects on *S. mutans*^28,29^. In this study, the highest concentration of a fluoride treatment was 2000 ppm, which was lower than those with reported bactericidal activity.

Polysaccharides account for up to 40% of the dry weights of dental biofilms, and they are typically synthesized by microbial glucosyltransferases^30^. Therefore, the reduction in biofilm biomass is directly related to the reduction of polysaccharide levels in the overall biofilm matrix. The complex structural integrity of a biofilm is mainly determined by the density of formation, volume, structural integrity, and stability, which are also associated with EPSs^7,31^. In this study, the effects of brief fluoride treatments on the synthesis of water-soluble/insoluble extracellular and intracellular polysaccharides during the formation of *S. mutans* biofilms were investigated. The synthesis of water-soluble/insoluble extracellular and intracellular polysaccharides in 46-h-old *S. mutans* biofilms was significantly inhibited in all fluoride treatment groups compared with the control group. The anti-EPS formation activity increased in a concentration-dependent manner (Fig. 1C–E; *p* < 0.05). In the 94-h-old *S. mutans* biofilms, only the 2000 ppm fluoride treatment group significantly inhibited the synthesis of water-insoluble extracellular and intracellular polysaccharides compared with the control group (Fig. 1C, p > 0.05; Fig. 1E, p < 0.05). In addition, the synthesis of water-soluble EPSs was significantly inhibited in the 1000 ppm and 2000 ppm fluoride treatment groups compared with the control group (Fig. 1D; *p* < 0.05).

Since the biofilms are mainly composed of bacterial cells and polysaccharides^7^, the decrease in dry weight in all fluoride treatment groups of the 46-h-old *S. mutans* biofilms and 2000 ppm fluoride treatment group of the 94-h-old *S. mutans* biofilms may be attributed to the decreased number of bacterial cells and suppressed EPS formation. The biovolumes and thicknesses of EPSs in *S. mutans* biofilms detected by confocal laser scanning microscopy also supported our findings. The formation of EPSs in biofilms was remarkably damaged by brief fluoride treatment, and the decrease in biovolumes and thicknesses of EPSs may further affect the growth height of bacterial cells (Fig. 5). Brief fluoride treatments inhibited the synthesis of biofilm polysaccharides, which was closely linked with the stage of biofilm formation and the concentrations of fluoride treatments. Compared with early-stage biofilms, a higher concentration of fluoride (≥ 1000 ppm) was required to significantly inhibit the synthesis of polysaccharides in the mature stages of cariogenic biofilms. In our study, the inhibitory capacity of the high concentration fluoride treatment group was higher than that of the low concentration fluoride treatment group. The inhibitory effects of brief fluoride treatments on polysaccharide syntheses in biofilms may be attributed to inhibition of glucosyltransferases activity or its production in *S. mutans* biofilm cells. The exact mechanism by which brief fluoride treatments inhibit glucosyltransferase activity and production in *S. mutans* biofilm cells, however, needs to be explored in the future.

Acid production by carbohydrates and acid tolerance in low pH environments are among the main toxicity characteristics of *S. mutans*^7^, which are closely associated with enamel demineralization and the formation of dental caries^32–34^. It is well known that fluoride has inhibitory effects on the acidogenicity of cariogenic biofilms^14^. In this study, the acid production and acid tolerance of *S. mutans* biofilm formation by brief fluoride treatment were studied. The results showed that fluoride treatments inhibited acid production and acid tolerance in *S. mutans* biofilm cells (Figs. 2, 3). In the pH changes of the old culture medium, it was found that all tested concentrations of fluoride treatment groups inhibited the acid-producing capacity of the biofilm in the early formation and mature stages of the biofilm. The concentration of acid in the old medium decreased in a concentration-dependent manner, and the highest inhibition ability was shown for the 2000 ppm fluoride treatment group (Fig. 2A,B). The decreases in acid concentrations may be related to decreases in biofilm cell viability or physiological capacity. In the glycolytic pH drop experiment, the initial pH value reflected the acid-producing capacity of the cell, and the final pH value reflected the acid-tolerance capacity of the cell^34^. As shown in Fig. 3, brief fluoride treatments inhibited acid production in the early and mature stages of cariogenic biofilms. The acidification activities of biofilms were inhibited in all fluoride treatment groups, and the 2000 ppm fluoride treatment group showed the lowest initial rate and total acid production (Fig. 3B,D). Additionally, the 2000 ppm fluoride treatment group showed reduced acid resistance of cariogenic biofilm cells, and it showed the highest final H^+^ concentration, indicating the most pronounced effect on the acid resistance of biofilm cells (Fig. 3A,C). The change in H^+^ concentration may be caused by the inhibition of glycolytic acid production by fluoride treatment. These results indicated that acid production and acid tolerance of the biofilm could be reduced by brief fluoride treatments performed during the early stage of biofilm formation and the mature stage. Anti-acidification activity was correlated with the concentration of fluoride, and the 2000 ppm fluoride treatment achieved the highest anti-acidification activity.

In general, brief fluoride treatments significantly inhibited the formation of *S. mutans* biofilms in the early stage. At the early stages of biofilm formation, the levels of dry weight, bacterial activity, water-soluble/insoluble polysaccharides, intracellular polysaccharides, and acid-producing capacity of *S. mutans* biofilms were significantly inhibited in all fluoride treatment groups compared with those of the control group (Figs. 1, 2, 3; *p* < 0.05). The inhibited growth of cariogenic bacteria in early-stage *S. mutans* biofilms was positively correlated with the concentration of fluoride treatment. At the mature biofilm stage, only the 2000 ppm fluoride treatment group significantly inhibited the growth of *S. mutans* biofilms compared with the control group. It is suggested that the inhibitory effects of brief fluoride treatments on the activity of *S. mutans* biofilms depend on the stage of biofilm formation. In the early stage of biofilm formation, fluoride treatment at a low concentration significantly inhibited the growth of biofilms, while a high concentration was required in the mature stage. Our experimental results also showed that the inhibitory effects of fluoride treatments on the growth of *S. mutans* biofilms either in the early or mature stage were related to the concentration of the fluoride treatment. Compared with other fluoride treatment groups and the control group, the 2000 ppm fluoride treatment group exhibited the strongest capability of inhibiting the growth of *S. mutans* biofilms. This study provides a theoretical basis for the timing of fluoride treatments used to prevent dental caries. However, further research is needed to reveal the exact mechanism underlying the role of fluoride treatments in inhibiting early-stage cariogenic biofilm formation.

## Conclusion

The antimicrobial activities of fluoride treatments on the growth of *S. mutans* biofilms were linked with the stage of biofilm formation. Brief fluoride treatments significantly inhibited the growth of early-stage *S. mutans* biofilms. The inhibition of *S. mutans* biofilm growth by fluoride treatment was easier in the early formation stage than in the mature stage. Fluoride treatment in the early stage of cariogenic biofilm formation may be an effective approach to controlling cariogenic biofilm development and preventing dental caries.

## Materials and methods

### Streptococcus* mutans* biofilms formation, fluoride, and experimental scheme

**Figure 6:**
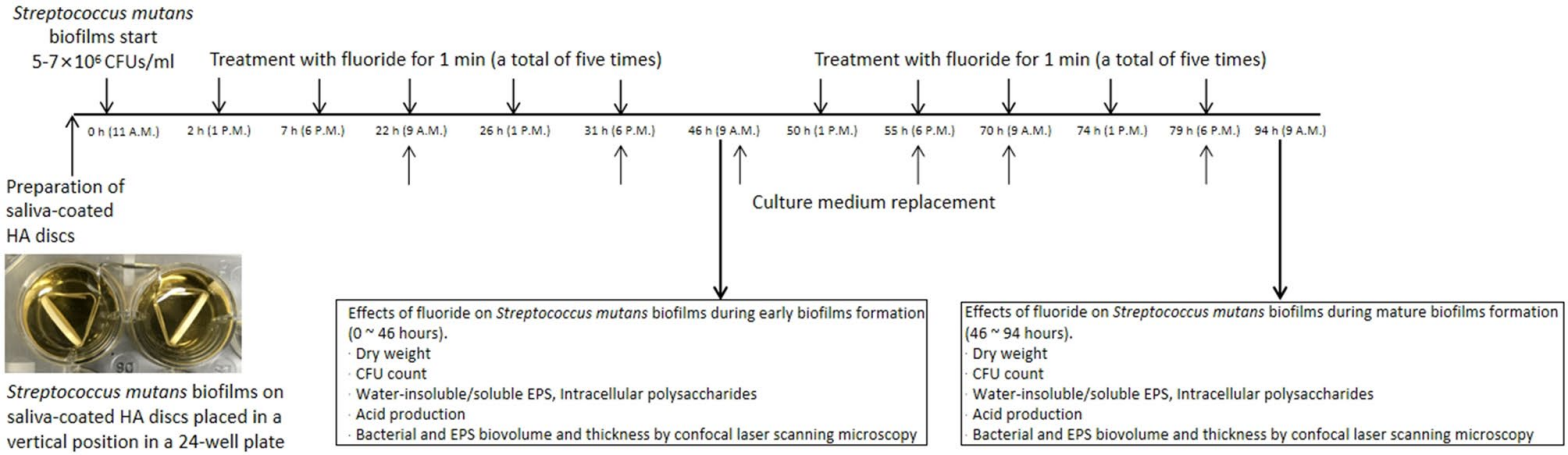
*Streptococcus mutans* biofilms formation and experimental scheme.

Figure 6 shows *S. mutans* biofilm preparation and experimental scheme for the present study. *S. mutans* UA159 (ATCC 700610; serotype c) biofilms were formed on saliva-coated hydroxyapatite discs (2.93 cm^2^; Clarkson Chromatography Products, Inc., South Williamsport, PA, USA) placed in a vertical position in 24-well plates. Briefly, an adult male was selected for oral saliva collection. hydroxyapatite discs were incubated in filter-sterilized (0.22-μm low protein-binding filter) saliva (3 ml/disc) for 1 h at 37 °C. For biofilms formation, the saliva-coated hydroxyapatite discs were transferred to a 24-well plate containing brain heart infusion (BHI; D-ifco, Detroit, MI, USA) broth with 1% (w/v) sucrose and *S. mutans* UA159 (5–7 × 10^6^ CFU/ml) (3 ml/disc). The biofilms were grown at 37 °C with 5% CO_2_. After 22 h of biofilm growth, the culture medium was changed twice daily (9 a.m. and 6 p.m.) until it was 46 h (0–46 h early biofilm formation) or 94 h (46–94 h mature biofilm formation)^10,15–17^. In the present study, the biofilms of ≥ 46 h were defined as mature biofilms^25,35^. This study is approved by the ethics committee/institutional review board of the Department of Preventive Dentistry, School of Dentistry, Institute of Oral Bioscience, Jeonbuk National University. All experimental protocols were approved by the Department of Preventive Dentistry, School of Dentistry, Institute of Oral Bioscience, Jeonbuk National University. The author confirms that all methods were carried out in accordance with relevant guidelines and regulations.

The fluoride source in this study was NaF. NaF was purchased from Sigma-Aldrich (St Louis, MO, USA). The solutions were made with NaF and purified water. Fluoride products at concentrations between 1 to 2000 ppm are recommended^8^. In this study, four concentrations of fluoride were used for the experiment, respectively: 250 ppm, 500 ppm, 1000 ppm, 2000 ppm.

To determine the anti-cariogenic biofilm activity of brief fluoride treatments during early *S. mutans* biofilm formation, the saliva-coated hydroxyapatite disks were treated with 0 ppm (control group), 250 ppm, 500 ppm, 1000 ppm, 2000 ppm for 1 min, a total of 5 times treated during the early *S. mutans* biofilm formation (at 2, 7, 22, 26, 31 h). The treated saliva-coated hydroxyapatite disks were washed with distilled water three times, then transferred into the original 24-well plates containing a 1% sucrose culture medium. The incubated time of the *S. mutans* biofilms was 46 h.

To determine the anti-cariogenic biofilm activity of brief fluoride treatments during mature *S. mutans* biofilms formation, *S. mutans* biofilms were not treated at the early biofilm growth stage. After mature biofilm formation, the saliva-coated hydroxyapatite disks were treated with 0, 250, 500, 1000, 2000 ppm for 1 min, a total of 5 times treated during the mature *S. mutans* biofilms formation (at 50, 55, 70, 74, 79 h). The treated saliva-coated hydroxyapatite disks were washed with distilled water three times, then transferred into the original 24-well plates containing a 1% sucrose culture medium. The incubated time of the *S. mutans* biofilms was 94 h.

The effects of different concentrations of fluoride on the dry weight, CFUs, water-soluble/insoluble EPSs, intracellular polysaccharide, and acidogenicity of early and mature biofilms were compared. The images of biofilms at 46-h-old and 94-h-old were obtained by confocal laser scanning microscopy.

### Microbiological and biochemical biofilm analyses

The dry weight and CFUs in the homogenized suspension were analyzed. Briefly, the 46/94-h-old biofilms on the saliva-coated hydroxyapatite disc were transferred into 2 ml of 0.89% NaCl and sonicated in an ultrasonic bath (Power sonic 410; Hwashin Technology Co., Seoul, Korea) for 10 min to disperse the biofilms. The dispersed solution was re-sonicated at 7 W for 30 s after adding 3 ml of 0.89% NaCl (VCX 130 PB; Sonics and Materials, Inc., Newtown, CT, USA). For the determination of CFUs count, an aliquot (0.1 ml) of the homogenized solution (5 ml) was serially diluted, plated onto brain heart infusion (BHI; Difco, Detroit, MI, USA) agar plates, and then incubated under aerobic conditions at 37 °C to determine the CFUs count^15,36^.

For the determination of the dry weight and amount of water-insoluble EPSs, water-soluble EPSs, intracellular polysaccharides, the remaining solution (4.9 ml) was centrifuged (3000×*g*) for 20 min at 4 °C. The biofilm pellet was resuspended and washed twice in the same volume of water. Mix the water washed the biofilms pellet with 95% alcohol and put it in a refrigerator at − 20 °C for at least 18 h to precipitate the water-soluble EPSs. Then calculated the content of water-soluble EPSs in the biofilms. The washed biofilms pellet is evenly divided into two portions, lyophilized, and weighed to determine the dry weight. One part used 1N sodium hydroxide to extract water-insoluble EPSs from the dried precipitate. The other part was used to calculate the content of intracellular polysaccharides, as detailed elsewhere^21^.

### Acid production analysis

The final pH values of the old culture media were also determined during the experimental period using a glass electrode (Beckman Coulter Inc., Brea, CA, USA) to investigate the change in acidogenicity of *S. mutans* biofilms by the treatments. The effect of brief fluoride treatment on the acidogenic and aciduric activity of early and mature *S. mutans* biofilms was determined by the glycolytic pH drop assay. Briefly, *S. mutans* biofilms were not treated with fluoride during the formation stage. the 46/94-h-old *S. mutans* biofilm was incubated in 20 mM potassium phosphate buffer (pH 7.2) for 1 h to deplete endogenous catabolites. They were then washed with salt solution (50 mM KCl + 1 mM MgCl_2_, pH 7.0) and treated with fluoride (0, 250, 500, 1000, 2000 ppm F−). After the fluoride treatment, the biofilms were dip-washed with salt solution and transferred into a 6-well plate containing salt solution. The pH was adjusted to 7.2 with a 0.2 M KOH solution. Glucose was then added to the mixture to give a final concentration of 1% (w/v). The decrease in pH was assessed using a glass electrode over 120 min (Futura Micro Combination pH electrode, 5 mm diameter; Beckman Coulter Inc., CA, USA). The effect of fluoride on the acid production of the biofilm was determined according to the acid production rate, calculated by the change in pH values over the linear portion (0–20, 30, 120 min) of the pH drop curves^37^.

The initial rate (0–20 min) of H^+^ production (y1) and initial rate (0–30 min) of H^+^ production (y2) was derived from the equation:

$$ {\text{y1 }} = \, \left( {{\text{H}}^{ + } {\text{concentration at 2}}0{\text{ min }}{-}{\text{ H}}^{ + } {\text{concentration at }}0{\text{ min}}} \right)/{2}0. $$

$$ {\text{y2 }} = \, \left( {{\text{H}}^{ + } {\text{concentration at 3}}0{\text{ min }}{-}{\text{ H}}^{ + } {\text{concentration at }}0{\text{ min}}} \right)/{3}0. $$

The total produced concentration of H^+^ (y3) was derived from the equation:

$$ {\text{y3 }} = {\text{ H}}^{ + } {\text{concentration at 12}}0{\text{ min }}{-}{\text{ H}}^{ + } {\text{concentration at }}0{\text{ min}}. $$

### Confocal laser scanning microscopy analysis

#### Live and dead bacterial cells staining

Confocal laser scanning microscopy analysis was performed to confirm the results of microbiological and biochemical studies. To investigate the difference in bacterial cells, the 46/94-h-old biofilms were stained at room temperature in the dark for 30 min using the Film Tracer LIVE/DEAD Biofilm viability kit L10316 (Invitrogen, Molecular Probes Inc., Eugene, OR, USA). The final concentrations of SYTO^®^9 and propidium iodide (PI) were 6.0 and 30 μM, respectively. This viability kit was based on plasma membrane integrity to determine live and dead cells. In this study, we regarded the cells with intact membranes (green) as live cells, whereas cells with damaged membranes (red) were regarded as dead cells. The excitation/emission wavelengths were 480/500 nm for SYTO^®^9 and 490/635 nm for PI for collecting the fluorescence. The stained live and dead bacterial cells were observed with an LSM 510 META microscope (Carl Zeiss, Jena, Germany) equipped with argon-ion and helium–neon lasers. All confocal fluorescence images were taken with an EC Plan-Neofuar 10x/0.30 M27 objective lens. A stack of slices in 6.4 μm step sizes was captured from the top to the bottom of the biofilms. The biovolume and thickness of live and dead cells were quantified from the entire stack using COMSTAT image-processing software. The biovolume is defined as the volume of the biomass (μm^3^) divided by the substratum (hydroxyapatite surface) area (μm^2^). The three-dimensional architecture of the biofilms was visualized using ZEN 2.3 (blue edition) (Carl Zeiss Microscopy GmbH, Jena, Germany). The original confocal data was uploaded to ZEN 2.3 software and the intensity of green and red fluorescence in the full thickness of biofilms layers were captured automatically. The software reconstructed the 2-dimensional intensity of fluorescence in all the layers to a 3-dimensional volume stack^38^.

#### EPS staining

The EPSs of 46/94-h-old biofilms were also investigated by simultaneous in situ labeling as described elsewhere^39^. Briefly, Alexa Fluor® 647-labeled dextran conjugate (1 μM, 10,000 MW; absorbance/fluorescence emission maxima 647/668 nm; Molecular Probes Inc., Eugene, OR, USA) was added to the culture medium during the formation of *S. mutans* biofilms (at 0, 22, 31 h of 46-h-old biofilms; at 0, 22, 31, 46, 55, 70, 79 h of 94-h-old biofilms) to label the newly formed EPSs. As described above, the stained EPSs were observed with an LSM 510 META microscope (Carl Zeiss, Jena, Germany) (objective: EC Plan Neofuar 10x/0.30 M27) equipped with argon-ion and helium–neon lasers and visualized using ZEN 2.3. A stack of slices in 7.8 μm step sizes was captured from the top to the bottom of the biofilms. Four independent experiments were performed, and five image stacks per experiment were collected. The EPSs biovolume and thickness were quantified from the confocal stacks using COMSTAT.

### Statistical analysis

All experiments (except CLSM and SEM) were performed in duplicate, and at least six different experiments were conducted. The data are presented as mean ± standard deviation. Inter-group differences were estimated using a one-way analysis of variance, followed by a post hoc multiple comparison (Tukey) test to compare multiple means (SPSS^®^ software, IBM). Values were considered statistically significant when the *p*-value was < 0.05.
